# Automated Grooming Detection of Mouse by Three-Dimensional Convolutional Neural Network

**DOI:** 10.3389/fnbeh.2022.797860

**Published:** 2022-02-02

**Authors:** Naoaki Sakamoto, Koji Kobayashi, Teruko Yamamoto, Sakura Masuko, Masahito Yamamoto, Takahisa Murata

**Affiliations:** ^1^Department of Animal Radiology, Graduate School of Agricultural and Life Sciences, The University of Tokyo, Tokyo, Japan; ^2^Autonomous Systems Engineering Laboratory, Graduate School of Information Science and Technology, Hokkaido University, Sapporo, Japan

**Keywords:** grooming, experimental animals, automated detection, mouse behavior, deep learning, convolutional neural network, 3D-CNN

## Abstract

Grooming is a common behavior for animals to care for their fur, maintain hygiene, and regulate body temperature. Since various factors, including stressors and genetic mutations, affect grooming quantitatively and qualitatively, the assessment of grooming is important to understand the status of experimental animals. However, current grooming detection methods are time-consuming, laborious, and require specialized equipment. In addition, they generally cannot discriminate grooming microstructures such as face washing and body licking. In this study, we aimed to develop an automated grooming detection method that can distinguish facial grooming from body grooming by image analysis using artificial intelligence. Mouse behavior was recorded using a standard hand camera. We carefully observed videos and labeled each time point as facial grooming, body grooming, and not grooming. We constructed a three-dimensional convolutional neural network (3D-CNN) and trained it using the labeled images. Since the output of the trained 3D-CNN included unlikely short grooming bouts and interruptions, we set posterior filters to remove them. The performance of the trained 3D-CNN and filters was evaluated using a first-look dataset that was not used for training. The sensitivity of facial and body grooming detection reached 81.3% and 91.9%, respectively. The positive predictive rates of facial and body grooming detection were 83.5% and 88.5%, respectively. The number of grooming bouts predicted by our method was highly correlated with human observations (face: *r* = 0.93, body: *r* = 0.98). These results highlight that our method has sufficient ability to distinguish facial grooming and body grooming in mice.

## Introduction

Experimental animals exhibit various behaviors, such as ambulation, immobility, rearing, scratching, and grooming. Since behavior reflects the mental and physical condition of an animal, we can estimate it by observing its behavior. Grooming is one of the common behaviors to care for fur, maintain hygiene, and regulate body temperature in experimental animals such as mice, rats, and others (Almeida et al., 2015; Kalueff et al., 2016). Grooming motion is composed of several microstructures, such as face washing and body licking (Kalueff and Tuohimaa, 2004; Kalueff et al., 2007). They typically groom themselves from the head to the genitals and tail. Several studies have shown that the internal status of rodents affects grooming frequency and/or duration. For example, stressors such as exposure to unfamiliar environments can elicit grooming behavior in mice (Kalueff and Tuohimaa, 2004; Reeves et al., 2016). Additionally, it has been discovered that ordered grooming microstructures are disturbed by neuropsychiatric and neurodegenerative disorders in mice (Berridge et al., 2005; Kalueff et al., 2016). These observations highlight the importance of quantitative and qualitative grooming assessments in the internal status evaluation of experimental animals.

There are several methods for assessing the grooming behavior of mice and rats. Human observation is a traditional and standard way to detect grooming. Although human observation does not require any specific equipment, it is labor-intensive and time-consuming. Additionally, the results vary between observers. Several automated methods have been developed to detect grooming in rodents. For example, Reeves et al. (2016) established the M-track, which tracks the position of the mouse forehand by painting fluorescent markers and detects grooming from its trajectories. Another group showed that the Janelia Automatic Animal Behavior Annotator, which is a machine learning classifier (Kabra et al., 2013), could detect grooming from top-recorded video files (van den Boom et al., 2017). Although these studies provide accurate and rapid grooming detection methods, many of them require special equipment. In addition, these systems did not distinguish grooming microstructures, such as face washing and body licking of mice and rats.

The development of deep neural network algorithms is noteworthy. Since Krizhevsky et al. (2012) showed that convolutional neural networks (CNNs) exhibit outstanding performance in image classification tasks, CNNs have become the *de facto* standard method for image recognition. CNN-based algorithms have also been developed in biology. For example, Pereira et al. (2019) developed LEAP, which estimates the pose of an animal from images. DeepLabCut is another common application for pose estimation using transfer learning (Mathis et al., 2018). We also recently showed that the combination of CNN and long short-term memory (LSTM) layers can be used to detect scratching behavior in mice, suggesting that deep neural networks can be applied for the detection of sequential movements (Kobayashi et al., 2021). Recently, Geuther et al. (2021) proposed an automated grooming detection method using a three-dimensional CNN (3D-CNN). However, a method using CNN to classify facial and body grooming has not been developed.

In this study, we aimed to establish a novel automated method to detect facial and body grooming in mice. Here, we showed that 3D-CNN can accurately classify facial and body grooming.

## Materials and Methods

### Mice Behavior Dataset

In this study, we used the videos recorded in the previous study to establish an automated scratching detection method (Kobayashi et al., 2021). Here, we briefly explain these videos. BALB/c mice (12–16 weeks old; male and female sex; *n* = 9) were treated with lysophosphatidic acid (LPA, Avanti Polar Lipids, Inc., Alabaster, AL, United States; 200 nmol/site/25 μl, 2 site/mouse, intracutaneously). Mice were then placed in a black square arena and their behavior was recorded by the hand camera (HDR-CX720V, Sony, Tokyo, Japan) set at a height of 150 cm above the arena. Recording conditions were as follows: frame rate, 60 Hz; resolution, 1,920 × 1,080 pixels, 24-bit color. We finally obtained 30 9-min video files and used them for grooming detection. We split the whole 30 videos into training (1–23), validation (24–25), and test (26–30) datasets. Detailed information is shown in Supplementary Table 1. We note that there is no overlap of mice between training and test datasets. Several neural networks were trained with the training dataset and evaluated their performance using the validation dataset. We finally chose the best model, and its performance was evaluated by the test dataset, which was used only here.

### Image Preprocessing and Integration

**FIGURE 1:**
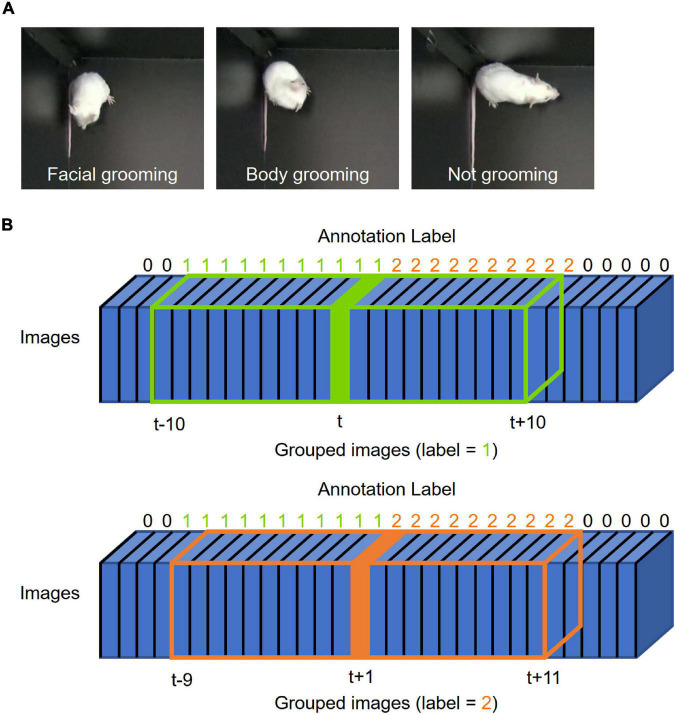
Grooming picture and image integration. **(A)** Representative pictures of grooming. The left, middle, and right pictures show representative images of facial grooming, body grooming, and not-grooming behavior, respectively. **(B)** Schematic illustration of “grouped images.” Blue boxes represent frame images. The number above each box indicates the label annotated by humans (0: not grooming, 1: facial grooming, 2: body grooming). The green box shows “grouped images at time *t*” integrated from *t*–10 to *t* + 10; the orange box shows “grouped images at time *t* + 1” integrated from *t*–9 to *t* + 11. The label at time *t* is used for that of “grouped images at time *t*” [blue; frame at time point *t* (label = 1), orange; frame at time point *t* + 1 (label = 2)].

We preprocessed images in a similar way to the previous study (Kobayashi et al., 2021) with slight modification. Briefly, images of all frames of each video were extracted, and an absolute difference between two sequential images was calculated. Differential images were cropped around the geometric center of the mouse into a square shape, resized to 128 × 128 pixels, and then gray-scaled and binarized. The geometric center of the mouse was obtained by a binary-image-based algorithm described in the previous study (Kobayashi et al., 2020). We integrated images at *t* ± 10, *t* ± 20, *t* ± 30, or *t* ± 40 for each time point *t* to use them for an input (Figure 1B), which were hereafter referred to as “grouped images.”

### Manual Grooming Annotation

We defined grooming as the following behavior which lasted for at least five frames and whose interval was at least six frames: washing face and head (facial grooming), licking or washing of paws, body, tail, and genital (body grooming). These thresholds were decided empirically during annotation processes. We carefully watched the videos and labeled each frame as “not grooming”: 0, “facial grooming”: 1, and “body grooming”: 2 (Figure 1A). The label of frame at time point *t* was assigned to the label of grouped images at *t* (Figure 1B). We note that at least two researchers checked the annotated labels and conflicts between them were resolved by discussion before training. Labels were converted to one-hot encoding vectors for training.

### The Architecture of Convolutional Recurrent Neural Network

**FIGURE 2:**
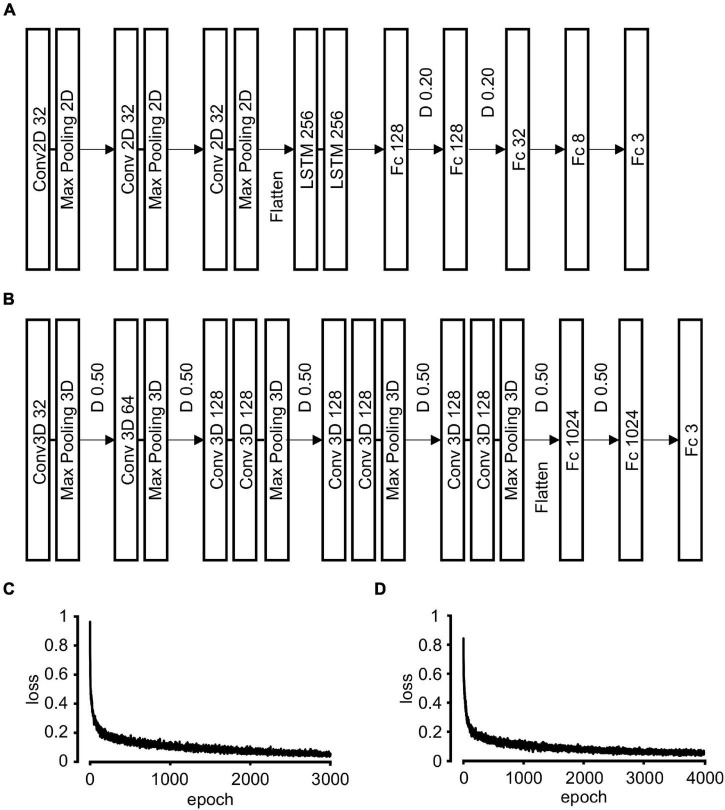
Neural network architecture and training. **(A)** Convolutional recurrent neural network (CRNN) architecture. The grouped images are input into three Conv2D and Max pooling 2D layers. The output tensor is flattened and integrated into two LSTM layers followed by five Fc layers. The number in each box refers to that of units in each layer. D 0.20: 20% drop out of the output units in each layer during training, Conv2D: 2D convolution, LSTM: long short-term memory, Fc: fully connected. **(B)** Three-dimensional convolutional neural network (3D-CNN) architecture. The grouped images are input into five Conv3D and Max pooling 3D layers. The output tensor was flattened and integrated into three Fc layers. The number in each box refers to that of units in each layer. D 0.50: 50% drop out of the output units in each layer during training, Conv3D: 3D convolution. **(C)** The transition of loss value during CRNN training. **(D)** The transition of loss value during 3D-CNN training.

The architecture and hyper-parameters of the convolutional recurrent neural network (CRNN) were almost similar to our previous study (Figure 2A) (Kobayashi et al., 2021). Briefly, the network was composed of three two-dimensional (2D)-CNN layers and two LSTM layers followed by five fully connected (Fc) layers. For the multiclass classification, we modified CRNN architecture as follows; the final layer has three nodes, and their activation function was softmax instead of sigmoid.

### The Architecture of Three-Dimensional Convolutional Neural Network

We constructed 3D-CNN having two blocks: 3D-CNN block and Fc block. We referred to the C3D model (Tran et al., 2015) in constructing our architecture. 3D-CNN blocks were composed of one or two 3D-convolutional layers [Conv3D; kernel size: (*t*, *w*, *h*) = (3, 3, 3) where *t* is temporal, *w* is width, and *h* is height axis) followed by 3D-max pooling layer [pooling size: (*t*, *w*, *h*) = (1, 2, 2) or (2, 2, 2)]. Output feature extracted by 3D-CNN block was flattened and input into Fc blocks. Fifty percent of units were randomly dropped out after each 3D-max pooling layer and between the first and second Fc layers. The activation function of the last layer was softmax and that of all the other layers was ReLU. Detailed information of layers is summarized in Figure 2B.

### Training of Neural Networks

Twenty-three videos (1–23) were used for the training of neural networks. We randomly reduced the number of grouped images labeled as not grooming. For one epoch, 1,000 of not grooming, 200 of facial grooming, and 400 of body grooming grouped images were randomly picked allowing duplicates (batch size was 8). Grouped images were randomly rotated by multiples of 20° and flipped for data augmentation. We used AMSGrad optimizer with 1 × 10^–4^ and 3 × 10^–5^ learning rates for CRNN training and 3D-CNN training, respectively. A categorical cross-entropy loss function was used for both trainings.

### Prediction of Neural Networks

The last layer of neural networks outputs three decimal values from 0 to 1 for each grouped image. These values were interpreted as the probability that the input grouped image belongs to three categories (i.e., not grooming, facial grooming, and body grooming). The category having the largest probability was adopted as the prediction of neural networks.

### Computer Hardware and Software

Trainings and predictions of neural networks were conducted on AI-COMPLIANT ADVANCED COMPUTER SYSTEM at the information initiative center, Hokkaido University, Sapporo, Japan, which was equipped with Intel Xeon Gold 6230 (2.1 GB) and NVIDIA Tesla V100 GPU (32 GB). Trainings and Predictions were conducted using the TensorFlow library (version 2.2.0) in Python.

## Results

### Video Capture and Image Preprocessing

In a previous study, we recorded the behavior of BALB/c mouse (12–16 weeks old; male and female sex; *n* = 9) and obtained 30 9-min video files (Kobayashi et al., 2021, Supplementary Table 1). Since these videos contained numerous grooming bouts, we reused these videos to establish an automated grooming detection method. For each video file, we obtained the frame images and preprocessed them as follows in order to reduce data size and remove background noise. First, differential images were obtained between two continuous frames. Then, these images were cropped and resized around the geometric center of the mouse into a square shape (128 × 128 pixels). Finally, the images were gray-scaled and binarized.

### Dataset Preparation of Grooming Behavior

We classified mouse grooming into two types, namely, “facial grooming” and “body grooming” (Figure 1A). Facial grooming was defined as washing face and head, while body grooming was defined as licking or washing of paws, body, tail, and genital. Each preprocessed image was labeled as “not grooming”: 0, “facial grooming”: 1, or “body grooming”: 2. Out of the 30 labeled videos, 23 (1–23) were used as the training dataset, two (24–25) were used as the validation dataset, and five (26–30) were used as the test dataset. In the training dataset, there were 659,797, 14,922, and 104,988 grouped images labeled as 0, 1, and 2, respectively. Because such an imbalanced dataset interferes with the efficient training of neural networks, we randomly selected 104,988 0-labeled images for training, which is identical to the number of 2-labeled images. In this study, we aimed to solve multiple classification problems (classifying each time point into 0, 1, or 2) using neural networks.

### Grooming Detection With Convolutional Recurrent Neural Network

First, we examined whether the CRNN-based algorithm, which was used for scratching detection in our previous study, could be applied to grooming detection. The architecture and parameters were modified to solve multiple classification problems (Figure 2A). We used *t* ± 10 grouped images for an input and trained CRNN with them (Figure 1B and Material and Methods).

During training, “loss,” an index of difference between predictions and labels, gradually declined and reached almost a plateau at 3,000 epochs (Figure 2C). We evaluated the performance of the CRNN at 3,000 epochs by predicting the labels in the training and validation datasets. For the training dataset, the sensitivity and positive predictive rate (PPR) of body grooming were 97.2% and 98.9%, respectively, and those of facial grooming were 98.4% and 90.1%, respectively (Table 1A). These results indicate that the CRNN was sufficiently trained. In contrast, for the validation dataset, the sensitivity and PPR of body grooming were 82.1% and 84.6%, respectively, and those of facial grooming were 71.4% and 72.1%, respectively (Table 1B). The accuracy, the ratio of correctly predicted frames, of CRNN was 96.5%, and the macro F1 score, the average of harmonic means of sensitivity and PPR for each class, was 0.844. Since sensitivity and PPR of facial grooming were about 70%, we aimed to improve the performance further.

**TABLE 1A:** Confusion matrix of convolutional recurrent neural network (CRNN) prediction for the training dataset.

Training dataset		Predicted label			Sensitivity
		Body	Face	Not grooming	
Human observation	Body	102038	1297	1653	97.2%
	Face	171	14688	63	98.4%
	Not	960	311	103717	
Positive predictive rate		98.9%	90.1%		

**TABLE 1B:** Confusion matrix of convolutional recurrent neural network (CRNN) prediction for the validation dataset.

Validation dataset		Predicted label			Sensitivity
		Body	Face	Not grooming	
Human observation	Body	5272	145	1004	82.1%
	Face	147	707	136	71.4%
	Not	815	129	59113	
Positive predictive rate		84.6%	72.1%		

### Grooming Detection With Three-Dimensional Convolutional Neural Network

We examined whether 3D-CNN could improve the discrimination ability of mouse grooming. We built an architecture of 3D-CNN (Figure 2B), referring to the C3D model (Tran et al., 2015). Here, *t* ± 40 grouped images were used for training. Loss gradually declined during training and reached a plateau at 3,000 epochs (Figure 2D). We evaluated the performance at 3,000 epochs by predicting the labels in the training and validation datasets. For the training dataset, the sensitivity and PPR of body grooming were 95.7% and 98.3%, respectively, and those of facial grooming were 90.4% and 92.4%, respectively (Table 2A). For the validation dataset, the sensitivity and PPR of body grooming were 87.2% and 90.2%, respectively, and those of facial grooming were 78.6% and 89.2%, respectively (Table 2B). The accuracy of 3D-CNN was 97.7%, and the macro F1 score was 0.904. We confirmed the superiority of 3D-CNN using the other five combinations of training/validation datasets (Supplementary Table 2). According to these results, we adopted 3D-CNN in the following experiments. We evaluated the trained 3D-CNN performance for the validation dataset at every 200 epochs and found that the accuracy and macro F1 scores also reached a plateau at 3,000 epochs (Supplementary Figures 1A,B). We also trained 3D-CNN using *t* ± 10, 20, or 30 grouped images for input; however, it did not improve the prediction performance (Supplementary Table 3). Based on these observations, we hereafter used the trained 3D-CNN with *t* ± 40 grouped images for 3,000 epochs.

**TABLE 2A:** Confusion matrix of three-dimensional convolutional neural network (3D-CNN) prediction for the training dataset.

Training dataset		Predicted label			Sensitivity
		Body	Face	Not grooming	
Human observation	Body	100500	913	3575	95.7%
	Face	732	13484	706	90.4%
	Not	991	202	103795	
Positive predictive rate		98.3%	92.4%		

**TABLE 2B:** Confusion matrix of three-dimensional convolutional neural network (3D-CNN) prediction for the validation dataset.

Validation dataset		Predicted label			Sensitivity
		Body	Face	Not grooming	
Human observation	Body	5601	40	780	87.2%
	Face	124	778	88	78.6%
	Not	486	54	59517	
Positive predictive rate		90.2%	89.2%		

### Filter Application to the Predicted Labels

**FIGURE 3:**
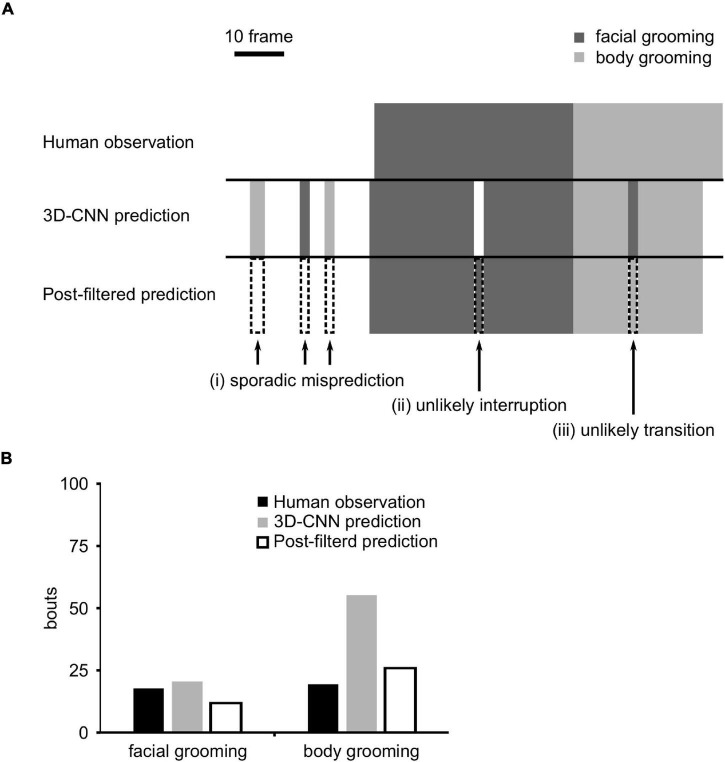
Filter application. **(A)** Schematic figure of three-dimensional convolutional neural network (3D-CNN) prediction and post-filtered prediction. Arrows indicate (i) sporadic prediction, (ii) unlikely interruption, or (iii) unlikely transition frames. The dotted box indicates the filtered frames. **(B)** The number of grooming bouts after the application of filters.

The prediction result of the 3D-CNN was carefully evaluated in the validation dataset and found that there were improbable predictions. We classified them into three patterns: (i) sporadic misprediction, (ii) unlikely interruption, and (iii) unlikely transition (Figure 3A). Sporadic mispredictions were defined as too short (≤ 4 frames) predictions of grooming. Unlikely interruptions were defined as overly short (≤ 6 frames) interruption sandwiched grooming bouts. Unlikely transitions were defined as too short (≤ 4 frames) grooming prediction before or after the other type of grooming. To exclude these predictions, we developed three filters corresponding to each one. These improbable predictions were serially reversed using filters (Figure 3A).

In the raw prediction data, the number of grooming bouts was overestimated owing to these improbable predictions (Figure 3B). Filter application remarkably decreased them and improved the number of predicted bouts (Figure 3B), although it did not affect PPR and sensitivity (Supplementary Table 4).

### Evaluation of the Trained Three-Dimensional Convolutional Neural Network and Filters

**FIGURE 4:**
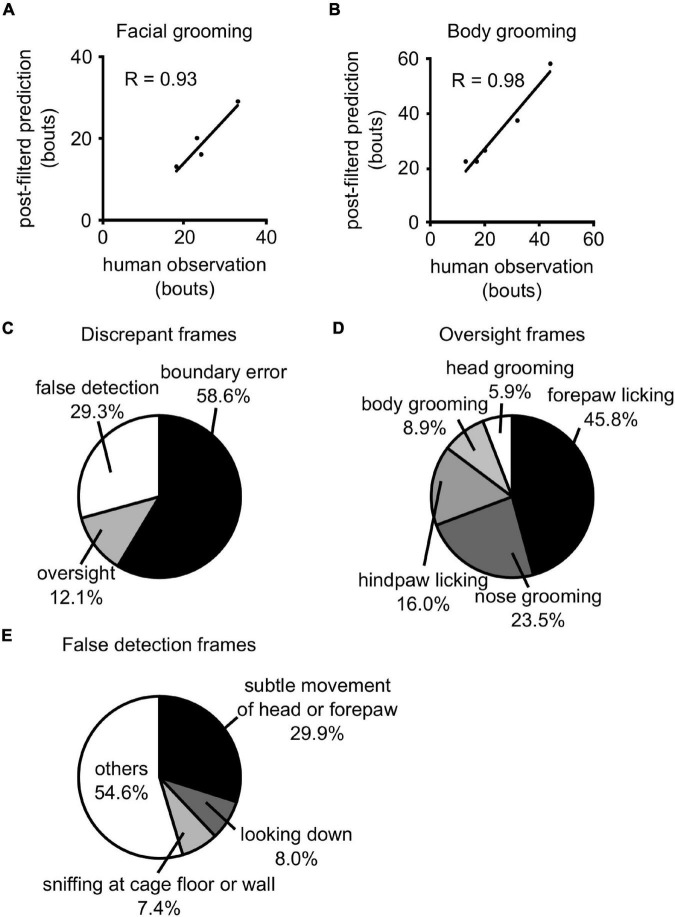
Prediction of the test dataset. **(A,B)** The comparison of the number of facial **(A)** and body **(B)** grooming bouts between human observation and post-filtered prediction. The lines indicate the regression line. *R*: correlation coefficient. **(C)** Details of discrepant frames between human observation and post-filtered prediction. **(D)** Details of oversight frames. **(E)** Details of false detection frames.

We then evaluated the performance of 3D-CNN and posterior filters for the test dataset, which has not been used up to here. After all filters were applied, the sensitivity and PPR of mouse body grooming were 91.9% and 88.5%, respectively, and those of facial grooming were 81.3% and 83.5%, respectively (Table 3). We also compared the number of facial and body bouts of post-filtered predictions with those of human observations. We found that the number of bouts of post-filtered predictions was comparable to that observed in humans (Figures 4A,B, face: *r* = 0.93; body: *r* = 0.98).

**TABLE 3:** Confusion matrix of post-filtered three-dimensional convolutional neural network (3D-CNN) prediction for the test dataset.

Post-filtered prediction		Predicted label			Sensitivity
		Body	Face	Not grooming	
Human observation	Body	21622	137	1757	91.9%
	Face	493	3268	261	81.3%
	Not	2328	507	139842	
Positive predictive rate		88.5%	83.5%		

### Evaluation of Errors in Post-filtered Predictions

We investigated the frame-by-frame differences between post-filtered predictions and human observations. Discrepant frames between them (false-positive and false-negative frames) were classified into three mutually exclusive error types: (i) boundary errors, (ii) oversights, and (iii) false detections. Boundary errors were defined as differences in the start and/or end of each grooming bout. Oversights were defined as bouts that were labeled as grooming by humans, although they were predicted as not grooming. False detections were defined as bouts that were incorrectly predicted as grooming. We found that boundary errors, oversights, and false detections accounted for 58.6%, 12.1%, and 29.3% of all discrepant frames, respectively (Figure 4C).

We also evaluated the types of behavior mice exhibited in oversights and false detection frames. We revealed that fine movements around the face were often overlooked (Figure 4D; forepaw licking: 45.8%, nose scratching: 23.5%). Additionally, subtle head or forepaw movements (29.9%), looking down (8.0%), and sniffing at the cage floor or wall (7.4%) accounted for a large portion of false detection frames (Figure 4E).

## Discussion

Since it is impossible to directly determine the mental and physical conditions of experimental animals, many researchers have paid attention to their behavior. We focused on grooming and successfully established a method to detect facial and body grooming in mice using 3D-CNN. Detailed evaluation of grooming has been focused on because various factors, including stressors, drug treatment, and genetic mutation, affect grooming frequency and pattern (Welch et al., 2007; Kalueff et al., 2016; Tartaglione et al., 2016). In particular, the regional distribution of grooming of rodents has been discovered to be worth analyzing. For example, rostral grooming has been reported to increase more than caudal grooming under stressed conditions (Kalueff and Tuohimaa, 2005). We classified grooming into facial and body grooming. In our datasets, mice spent more time body grooming than facial grooming. This grooming distribution was similar to that of rats splashed with water, which spent more time body grooming than facial grooming (van Erp et al., 1994; Shiota et al., 2016). Here, we established a novel method to classify facial and body grooming, which enabled us to analyze grooming microstructure in an objective, high-throughput manner.

An automated grooming detection system is required as human observation is labor-intensive and has low throughput. An automated system is essential to be convenient and to have sufficient performance compared with human observations. In this study, we developed a novel automated grooming classifier composed of a commercially available hand camera, normal home cage, and GPU-mounted computer. This simplicity is superior to existing methods, which often require special equipment. More importantly, our system achieved sufficient performance in distinguishing facial and body grooming after the application of filters (Table 3). Analysis of differences between human observation and post-filtered prediction showed that more than half of the discrepant frames were boundary errors, which are differences regarding the start and/or end of each grooming bout. This error can occur among human observations, which is a non-specific problem in our system. Although the left errors were indeed faults of our system, such error frames were few enough compared with those of accurately detected frames. Therefore, our system has sufficient discrimination ability for facial and body grooming with only top-view images. These results highlight the superiority of the proposed method.

Two-dimensional (2D)-CNN has become the *de facto* standard method of image recognition in many research fields, including animal ethology. Recently, to treat time-series data effectively, we and others combined 2D-CNN and RNN and succeeded in detecting animal behavior, including scratching in mice (Kobayashi et al., 2021) and daily behavior in cows (Wu et al., 2021). We also attempted CRNN to detect grooming; however, its performance was not sufficient in this study (Table 1B). Here, we applied another architecture, 3D-CNN, which can deal with a series of planar images as the cubic object. This architecture exhibits excellent performance in detecting facial and body grooming. Recently, Geuther et al. (2021) also proposed a 3D-CNN-based grooming detection system (grooming vs. not grooming). Our results were consistent with their results and also highlighted the effectiveness of 3D-CNN for facial and body grooming discrimination.

This study has the following limitations. First, our 3D-CNN was not suitable for detecting fine movements (Figures 4D,E). It is sometimes difficult even for human observers to distinguish grooming from unrelated behavior as we have only top-view images that have blind spots. For example, forepaw licking and unrelated subtle movements of the head or forepaws are ambiguous. We assumed that multi-angle recording may improve accuracy in both human annotation and prediction. Second, whether our 3D-CNN can be used in other datasets has not been validated. Since we used binarized differential images between consecutive frames for the input of neural networks, our method does not depend on the colors of mouse fur in theory. However, it is possible that mouse size or light conditions in the recording environment affect the performance. Third, hyper-parameters such as data downsizing ratio and class ratio were not fully optimized. Their further optimization would improve the performance. Finally, our architecture, simple 3D-CNN, was not the state-of-the-art method. As deep learning methods have been rapidly evolved, many architectures have been continuously proposed (Qiu et al., 2017; Feichtenhofer et al., 2019; Fan et al., 2021). Additionally, there are often cases that pre-trained models were disclosed. Further application of these technologies can improve the discrimination ability.

In conclusion, we developed a 3D-CNN-based grooming detection method that can automatically distinguish facial and body grooming.

## Data Availability Statement

The raw data supporting the conclusions of this article will be made available by the corresponding author, without undue reservation.

## Ethics Statement

All experiments were reviewed and approved by the institutional Animal Care and Use Committee at the University of Tokyo (P19–079). Animal care and treatments were performed in accordance with the guidelines outlined within the Guide to Animal Use and Care of the University of Tokyo.

## Author Contributions

NS, KK, MY, and TM contributed to the conception and design of the study. NS, KK, TY, and SM performed the experiments. NS and KK analyzed the data. NS wrote the first draft of the manuscript. NS, KK, and TM revised the manuscript. All authors read and approved the submitted version.

## Conflict of Interest

The authors declare that the research was conducted in the absence of any commercial or financial relationships that could be construed as a potential conflict of interest.

## Publisher’s Note

All claims expressed in this article are solely those of the authors and do not necessarily represent those of their affiliated organizations, or those of the publisher, the editors and the reviewers. Any product that may be evaluated in this article, or claim that may be made by its manufacturer, is not guaranteed or endorsed by the publisher.

## References

[B1] Almeida M. C. Vizin R. C. L. Carrettiero D. C. (2015). Current understanding on the neurophysiology of behavioral thermoregulation. *Temperature* 2 483–490. 10.1080/23328940.2015.1095270 27227068 PMC4843931

[B2] Berridge K. C. Aldridge J. W. Houchard K. R. Zhuang X. (2005). Sequential super-stereotypy of an instinctive fixed action pattern in hyper-dopaminergic mutant mice: a model of obsessive compulsive disorder and Tourette’s. *BMC Biol.* 3:4. 10.1186/1741-7007-3-4 15710042 PMC552313

[B3] Fan H. Xiong B. Mangalam K. Li Y. Yan Z. Malik J. (2021). *Multiscale Vision Transformers.* Available Online at: http://arxiv.org/abs/2104.11227 (accessed April 22, 2021).

[B4] Feichtenhofer C. Fan H. Malik J. He K. (2019). “Slowfast networks for video recognition,” in *Proceedings of the IEEE International Conference Computer Vision*, (Piscataway, NJ: IEEE). 10.3390/s20082381

[B5] Geuther B. Q. Peer A. He H. Sabnis G. Philip V. M. Kumar V. (2021). Action detection using a neural network elucidates the genetics of mouse grooming behavior. *Elife* 10:e63207. 10.7554/eLife.63207 33729153 PMC8043749

[B6] Kabra M. Robie A. A. Rivera-Alba M. Branson S. Branson K. (2013). JAABA: Interactive machine learning for automatic annotation of animal behavior. *Nat. Methods* 10 64–67. 10.1038/nmeth.2281 23202433

[B7] Kalueff A. V. Tuohimaa P. (2004). Grooming analysis algorithm for neurobehavioural stress research. *Brain Res. Protoc.* 13 151–158. 10.1016/j.brainresprot.2004.04.002 15296852

[B8] Kalueff A. V. Tuohimaa P. (2005). The grooming analysis algorithm discriminates between different levels of anxiety in rats: potential utility for neurobehavioural stress research. *J. Neurosci. Methods* 143 169–177. 10.1016/j.jneumeth.2004.10.001 15814150

[B9] Kalueff A. V. Stewart A. M. Song C. Berridge K. C. Graybiel A. M. Fentress J. C. (2016). Neurobiology of rodent self-grooming and its value for translational neuroscience. *Nat. Rev. Neurosci.* 17 45–59. 10.1038/nrn.2015.8 26675822 PMC4840777

[B10] Kalueff A. V. Wayne Aldridge J. Laporte J. L. Murphy D. L. Tuohimaa P. (2007). Analyzing grooming microstructure in neurobehavioral experiments. *Nat. Protoc.* 2 2538–2544. 10.1038/nprot.2007.367 17947996

[B11] Kobayashi K. Matsushita S. Shimizu N. Masuko S. Yamamoto M. Murata T. (2021). Automated detection of mouse scratching behaviour using convolutional recurrent neural network. *Sci. Rep.* 11:658. 10.1038/s41598-020-79965-w 33436724 PMC7803777

[B12] Kobayashi K. Shimizu N. Matsushita S. Murata T. (2020). The assessment of mouse spontaneous locomotor activity using motion picture. *J. Pharmacol. Sci.* 143 83–88. 10.1016/j.jphs.2020.02.003 32178942

[B13] Krizhevsky A. Sutskever I. Hinton G. E. (2012). “ImageNet classification with deep convolutional neural networks,” in *Proceedings of the 25th International Conference on Neural Information Processing Systems NIPS’12*, Vol. 1, 1097–1105.

[B14] Mathis A. Mamidanna P. Cury K. M. Abe T. Murthy V. N. Mathis M. W. (2018). DeepLabCut: markerless pose estimation of user-defined body parts with deep learning. *Nat. Neurosci.* 21 1281–1289. 10.1038/s41593-018-0209-y 30127430

[B15] Pereira T. D. Aldarondo D. E. Willmore L. Kislin M. Wang S. S. H. Murthy M. (2019). Fast animal pose estimation using deep neural networks. *Nat. Methods* 16, 117–125. 10.1038/s41592-018-0234-5 30573820 PMC6899221

[B16] Qiu Z. Yao T. Mei T. (2017). “Learning spatio-temporal representation with pseudo-3D residual networks,” in *Proceedings of the IEEE International Conference Computer Vision*, (Piscataway, NJ: IEEE).

[B17] Reeves S. L. Fleming K. E. Zhang L. Scimemi A. (2016). M-Track: a new software for automated detection of grooming trajectories in mice. *PLoS Comput. Biol.* 12:1005115. 10.1371/journal.pcbi.1005115 27636358 PMC5026371

[B18] Shiota N. Narikiyo K. Masuda A. Aou S. (2016). Water spray-induced grooming is negatively correlated with depressive behavior in the forced swimming test in rats. *J. Physiol. Sci.* 66 265–273. 10.1007/s12576-015-0424-1 26586000 PMC10717009

[B19] Tartaglione A. M. Armida M. Potenza R. L. Pezzola A. Popoli P. Calamandrei G. (2016). Aberrant self-grooming as early marker of motor dysfunction in a rat model of Huntington’s disease. *Behav. Brain Res.* 313 53–57. 10.1016/j.bbr.2016.06.058 27374158

[B20] Tran D. Bourdev L. Fergus R. Torresani L. Paluri M. (2015). “Learning spatiotemporal features with 3D convolutional networks,” in *Proceedings of the IEEE International Conference Computer Vision*, (Piscataway, NJ: IEEE).

[B21] van den Boom B. J. G. Pavlidi P. Wolf C. J. H. Mooij A. H. Willuhn I. (2017). Automated classification of self-grooming in mice using open-source software. *J. Neurosci. Methods* 289 48–56. 10.1016/j.jneumeth.2017.05.026 28648717

[B22] van Erp A. M. M. Kruk M. R. Meelis W. Willekens-Bramer D. C. (1994). Effect of environmental stressors on time course, variability and form of self-grooming in the rat: handling, social contact, defeat, novelty, restraint and fur moistening. *Behav. Brain Res.* 65 47–55. 10.1016/0166-4328(94)90072-8 7880454

[B23] Welch J. M. Lu J. Rodriguiz R. M. Trotta N. C. Peca J. Ding J. D. (2007). Cortico-striatal synaptic defects and OCD-like behaviours in Sapap3-mutant mice. *Nature* 448 894–900. 10.1038/nature06104 17713528 PMC2442572

[B24] Wu D. Wang Y. Han M. Song L. Shang Y. Zhang X. (2021). Using a CNN-LSTM for basic behaviors detection of a single dairy cow in a complex environment. *Comput. Electron. Agric.* 182:106016. 10.1016/j.compag.2021.106016

