# Supplementary material for: Automated Grooming Detection of Mouse by Three-Dimensional Convolutional Neural Network
Source: Front Behav Neurosci. 2022 Feb 2;16:797860. doi: 10.3389/fnbeh.2022.797860 (PMC8847608; doi:10.3389/fnbeh.2022.797860)
Supplement: Supplementary file 1 [file Data_Sheet_1.PDF]

Supplementary Material

1 Supplementary Figures

Fig S1

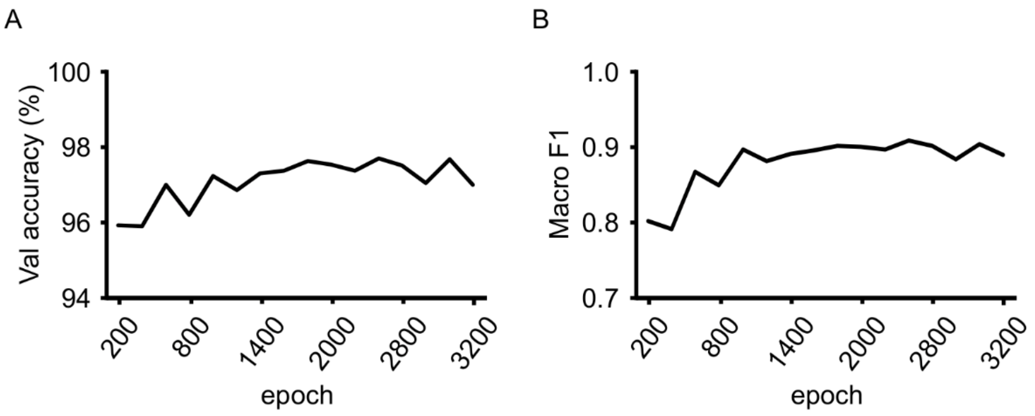

Fig. S1 The performances of 3D-CNN trained for different epochs

(A and B) The transition of validation accuracy **(A)** and Macro F1 score **(B)**

2 Supplementary Tables

Table S1 Dataset information

	Video No.	Mouse No.	Mouse sex	time (min:sec)
Training	01	A	male	9:49
	02	A	male	9:23
	03	A	male	9:09
	04	B	female	9:24
	05	B	female	9:18
	06	B	female	9:23
	07	D	male	9:27
	08	D	male	9:26
	09	D	male	9:15
	10	E	male	10:21
	11	E	male	9:27
	12	F	male	9:15
	13	F	male	9:29
	14	F	male	9:16
	15	F	male	9:19
	16	G	male	9:17
	17	G	male	9:45
	18	G	male	9:25
	19	G	male	9:21
	20	G	male	9:21
	21	H	male	9:26
	22	H	male	9:42
	23	H	male	9:27
Validation	24	H	male	9:27
	25	I	male	9:21
Test	26	I	male	10:01
	27	I	male	9:23
	28	J	male	9:23
	29	J	male	9:20
	30	J	male	9:21

Table S2 The comparison of the performance between 3D-CNN and CRNN.

	3D-CNN	CRNN
accuracy	97.22±0.97*	96.42±1.16
face sensitivity	77.71±12.44	74.12±5.31
body sensitivity	89.63±4.31	86.56±6.87
face PPR	79.81±10.71*	70.75±9.74
body PPR	85.57±7.09	82.48±9.30
macro F1	0.8799±0.0441*	0.8477±0.0421

* $p<0.05$ by paired t test compared with CRNN

Video No. (1, 2), (3, 4), (5, 6), (7, 8), (9, 10), or (24, 25) were used for the validation dataset respectively, and other videos were used for the training dataset (e.g., validation: No. 3 and 4, training: No. 1, 2, and 5-25). Data are presented as mean ± SD.

Table S3 Comparison of the performance using different size of grouped images

	accuracy	sensitivity		PPR		MacroF1 score
		facial	body	facial	body	
t ± 10	96.7%	50.1%	80.5%	94.5%	88.9%	0.828
t ± 20	97.4%	75.7%	89.1%	90.2%	86.2%	0.895
t ± 30	97.4%	77.8%	87.4%	90.3%	87.6%	0.899
t ± 40	97.7%	78.6%	87.2%	89.2%	90.2%	0.904

Table S4 Confusion matrix of post-filtered 3D-CNN prediction for the validation dataset.

Validation dataset post-filtered results		predicted label			sensitivity
		Body	face	not grooming	
human observation	body	5590	32	799	87.1%
	face	128	775	87	78.3%
	not	462	60	59535	
Positive predictive rate		90.5%	89.4%		
